# The Impact of a Neurocollaborative Theraplay-informed Intervention on the Presentations of Developmental Trauma and Attachment Difficulties in Attention Deficit Hyperactivity Disorder Children: A Pilot Study

**DOI:** 10.1007/s40653-026-00871-w

**Published:** 2026-04-29

**Authors:** Jack Purrington, Rhea Mistry, Robert Johnson, Izzy Rose, Alan D Price, Chloe Godfrey, Jacqueline Lynch, Raja A S Mukherjee

**Affiliations:** 1 Chrysalis Associates, Sheffield, UK; 2 University of Salford, Salford, United Kingdom; 3 Surrey and Borders Partnership NHS Foundation Trust, Leatherhead, United Kingdom

**Keywords:** ADHD, Trauma, Attachment disruption, Theraplay

## Abstract

The overlapping symptoms of ADHD, developmental trauma, and attachment difficulties in children complicate accurate assessment and intervention. Despite this, limited research has explored the efficacy of Theraplay for children with experiences of both ADHD and developmental trauma. This study evaluated the efficacy of a 30-hour, neuro-collaborative, Theraplay-informed intervention for looked-after children with ADHD, comparing outcomes to a non-ADHD control group. A mixed design was implemented, with two groups (ADHD and non-ADHD; *N* = 14 per group) participating between March 2019 and March 2024. The intervention included 10 h of therapeutic parenting based on Theraplay parent sessions and principles of dyadic developmental parenting, 10 h of family therapy based on the principles of Theraplay, and 10 h of sensory regulation work tailored to the child or family unit. Pre- and post-intervention outcomes were measured using the Assessment Checklist for Children (ACC), Behaviour Rating Inventory of Executive Functioning – 2nd Edition (BRIEF-2), Child Behaviour Checklist (CBCL), and Trauma Symptom Checklist for Young Children (TSCYC). Paired t-tests in the ADHD group revealed significant post-intervention reductions in CBCL total problems, rule-breaking behaviour, oppositional defiance, and TSCYC anxiety scores. Analysis of Covariance (ANCOVA) results found no significant differences between the ADHD and non-ADHD groups when controlling age, and gender, pre-test scores as covariates and post-test scores as the dependent variable. These findings suggest positive therapeutic outcomes for ADHD children with an improvement rate similar to their matched controls.

**Supplementary Information:**

The online version contains supplementary material available at https://doi.org/10.1007/s40653-026-00871-w.

Attention Deficit Hyperactivity Disorder (ADHD) is defined by the American Psychiatric Association (APA) as a neurodevelopmental condition characterised by impaired levels of inattention, disorganisation, and/or hyperactivity-impulsivity (APA, 2013; 2022; Koutsoklenis & Honkasilta, 2023). In the UK, the National Institute for Health and Care Excellence (NICE) outlines that ADHD symptoms include at least six of nine inattentive symptoms and/or at least six of nine hyperactivity-impulsivity symptoms.

ADHD can be categorised into three main subtypes: (1) predominantly inattentive, (2) predominantly hyperactivity, and (3) combined presentations. The NICE (2023) guidelines recommend that ADHD should be suspected if children exhibit at least six inattentive symptoms and/or at least six hyperactivity-impulsivity symptoms with these symptoms needing to have started prior to the age of 12 years, to occur in two or more settings such as home and school, to be present for greater than 6 months, to clearly interfere with, or reduce the quality of social, academic, or occupational functioning, and to be independent to psychotic experiences or not be better explained by another conceptualised experience such as oppositional defiance or conduct difficulties.

The estimated prevalence of ADHD within school-aged children has been seen to be between 4% and 12% worldwide (Polancyk et al., 2007), although NICE (2019) provide a range between 3% and 9% and add that this may increase due to revised criteria. There is therefore a real need to develop a comprehensive understanding of best practice within assessment, diagnosis, and management pathways for children and young people. Current NICE (2019) guidelines for the diagnosis and management for ADHD in children recommend the following:

1. For children under the age of 5 years: a group-based parent-training programme and environmental modifications as first-line treatments.
2. For children aged 5 years and over: group-based ADHD-focused support and environmental modifications, group or individual parent-training programmes, followed by medication if symptoms persist, followed by cognitive behavioural therapy if symptoms persist.

These guidelines are recommended within the context that if a child presents with oppositional defiant disorder (ODD) or conduct disorder (CD) symptom clusters parents should be offered parent-training programs in line with NICE’s guidelines on antisocial behaviour and conduct disorders (NICE, 2019). Whilst parent training is an evidenced-based treatment for ADHD across age groups, the NICE recommendation is framed separately which implies that recommendations for ADHD and ODD or CD are portrayed as mutually exclusive recommendations as opposed to potentially concurrently complementary. However, there are several overlapping symptoms between ADHD and other conditions impacting diagnostic decisions such as developmental trauma (Collins & Hartley, 2023), that may benefit from a broader, more integrative approach when both experiences co-exist.

For the purpose of this pilot study developmental trauma is utilised to described the unique traumatic response type often seen in children who have experienced emotional, sexual, or physical abuse and/or neglect and/or domestic violence perpetrated by a caregiver within a primary attachment relationship (van der Kolk, 2005). Traumatic experiences that take place within primary attachment relationships can often result in attachment difficulties and at times attachment disruption. Attachment disruption refers to the experience of being separated from a primary attachment relationship which often contributes to the development of insecure or disorganised attachment patterns (NICE, 2015).

In England, Department for Education ([DfE], 2023) data suggests that approximately 94% of children currently in care were removed from their primary caregivers due to experiences of absent parenting, family in acute stress, family dysfunction, or abuse/neglect. A child in care is a child who has been looked after by the local authority for more than 24 h, where legal responsibility has been given by a court to adoptive parents, a special guardian, a foster carer, or a parent. All of these experiences are synonymous with the prodromal stages of developmental trauma and attachment difficulties. Subsequently, care experienced adoptive children often present with varying levels of trauma and attachment symptomology relating to their previous experiences.

When developmental trauma is experienced by an individual who also possesses certain pre-existing physiological vulnerabilities, the development of heightened neurobiological dysregulations may then be observed post-trauma exposure; such vulnerabilities can include an inherently reduced prefrontal inhibitory control, innate differences in HPA axis responsivity and low baseline dopaminergic regulation (Lanius et al., 2010). This is an interpretation of the Stress-Diathesis model (Broerman, 2017). These vulnerabilities may be in the form of genetic factors governing brain-derived neurotrophic factors, for example, which has been shown to effect resilience and stress-sensitivity (Notaras & van den Buuse, 2020).

The impact of developmental trauma alongside pre-existing vulnerabilities can result in a wide range of neurobiological adaptations (Perry et al., 1995). Many of these adaptations can result in behavioural presentations that appear to mirror presentations of children with ADHD. For example, developmental trauma experiences can lead to the dysregulation of the hypothalamic pituitary adrenocortical axis (Bunea et al., 2017), responsible for regulating the body’s response to stress. This can result in children who appear inattentive and/or hyperactive and impulsive as a result of being activated into survival responses. Equally, studies have found that developmental trauma can result in reduced and/or dysregulated activity in the cerebellum (Teicher et al., 2006), orbitofrontal cortex and anterior cingulate gyrus (Chugani et al., 2001). The former playing a role in language and attention and the latter two contributing to decision making, inhibition, and attention. Dysregulated activity in these brain regions can therefore contribute to behavioural presentations that can again appear inattentive and/or hyperactive and impulsive.

There is a cohort of care experienced children that have experienced developmental trauma, attachment disruption, and have a diagnosis of ADHD. Recent scoping and systematic reviews exploring the relationship between ADHD and childhood trauma have highlighted that the two experiences have an additive effect on each other, in that ADHD worsens the severity of childhood trauma symptomatology and childhood trauma worsens the severity of ADHD symptomatology (Boodoo et al., 2022; Craig et al., 2020). Additionally, literature exploring attachment experiences across wider neurodiverse cohorts such as Autistic children and children with Fetal Alcohol Spectrum Disorder (FASD) have found that attachment experiences can be influenced by neurodivergence, separate to developmental trauma experiences (Martinez, 2024; Rutgers et al., 2004). The unique impact of co-occurring developmental trauma, attachment disruption, and ADHD highlights the need for research into efficient and effective psychological interventions for looked after children and their families when these difficulties occur together.

Several play therapy approaches such as child-centred play therapy, filial therapy, parent-child interaction therapy, and therapeutic play are commonly used to support children with trauma and attachment difficulties. Many play therapies rely on verbal reflection or symbolic play, which can be challenging for children with the unique intersection of developmental trauma, attachment difficulties, and neurodiversity. One therapeutic approach that has shown unique promise for neurodiverse care experienced children who have experienced developmental trauma and attachment disruption is Theraplay^®^. Theraplay differs from child-led or symbolic and interpretive play therapies by emphasising structured, adult-guided, multi-sensory, and here-and-now interactions that strengthen child-caregiver relationships through direct experiences of safety, connection, and co-regulation.

Theraplay is described as “an engaging, playful, relationship-focused treatment method that is interactive, physical, and fun…” (Booth & Jernberg, 2010, p. xxi) which “…uses attachment-based play to create better relationships between parents and their children” (Booth & Koller, 1998: p. 308).

Theraplay involves the use of nine core concepts which can be used to flexibly and adaptively meet the presenting needs of children and families during therapy. These core concepts are that Theraplay is: multi-sensory; guided by the adults; culturally sensitive; playful; preverbal, social, and right-brained; co-regulated; responsive, attuned, empathic, and reflective; interactive relationship-based; and a direct here-and-now experience. Through these core conditions activities take place within four dimensions of Engagement (focusing on connection, optimal arousal, and shared joy), Nurture (focusing on self-worth, empathy, and positive touch), Structure (focusing on safety, organisation, and regulation), and Challenge (focusing on competence, confidence, and growth). These experiential activities aim to strengthen attachments, promote positive mental health, develop self-regulation, build social skills, nurture positive internal working models, and encourage the ability to learn.

Research exploring the Theraplay approach has found the model to be effective in improving behavioural regulation and functioning (Francis et al., 2017; Purrington et al., 2022; Rezaeianzadeh & Yazdanfar, 2024; Weir et al., 2013), improving affect regulation and internalised symptoms (Lindaman & Lender, 2009; Siu, 2009), and enhancing interpersonal relationships and sociality (Weir et al.,^2020^; Wettig et al., 2011). These conclusions are mirrored in recent systematic (Money et al., 2021; Purrington, Goodall et al., 2023), scoping (France et al., 2023), and expert (Purrington, Lindaman, & Jernberg, 2025) reviews of Theraplay and post-adoption support interventions which conclude that there is promising evidence to suggest that Theraplay can reduce behavioural and emotional challenges and that Theraplay can be utilised with a range of presenting difficulties, predominantly resulting in positive post-therapy outcomes.

In addition, Theraplay has also been utilised with a number of neurodiverse populations including Autistic children (Chang et al., 2021; Coleman & Hong, 2023; Goodyear-Brown, 2009; Hiles-Howard et al., 2018; Simeone-Russell, 2011), children who meet the threshold for an intellectual disability (Siu, 2014; Hofstra et al., 2023; Mohamed & Mkabile, 2015), and children prenatally exposed to alcohol (PAE) with suspected Fetal Alcohol Spectrum Disorder (FASD; Purrington, Price, et al. 2025. For families with children with neurodiversity and experiences of developmental trauma and attachment disruption, a recent scoping review further corroborates the use of Theraplay; Kalisch et al. (2023) found that for neurodiverse children with interpersonal trauma experiences the most promising intervention approaches include those that prioritise parent and caregiver involvement and place less of an emphasis on verbal skills, much like Theraplay.

Explorations of the use of Theraplay with children with ADHD include a clinical case vignette presented by Kiermaier (2010); a clinical practice discussion by Siu (2017); a single case study report by Sundberg et al. (2017) which details the impact of 35 sessions of Theraplay with a child who started therapy aged 5 with their biological parents; and Askari et al. (2014) which found 10 sessions of Theraplay to be more successful than behaviour management training in reducing symptoms of ADHD in a sample of 60 non-looked after children with ADHD aged between 7 and 12 years old. ADHD is also discussed in relation to Theraplay by Wettig et al. (2011) however, is not the primary focus on the study. Despite these tentative explorations there is yet to be a study exploring the impact of Theraplay for care experienced children who have ADHD and have experienced developmental trauma and attachment disruptions, this is despite clear understanding of the additive effect of the co-occurrence of childhood trauma and ADHD, each making the other more complex.

Therefore, the purpose of this pilot study was to review the efficacy of a 30-hour neuro-collaborative Theraplay-informed psychotherapeutic intervention on the presentations of developmental trauma and attachment difficulties in looked after children with ADHD who have experienced developmental trauma and attachment disruption.

## Method

### Ethics

The University of Salford provided ethical approval for this study; ethical approval reference 3171. Furthermore, all procedures adhered to the British Psychological Society’s code of ethics and conduct and the Health and Care Professionals Council standards of conduct performance and ethics. Additionally, prior to commencing therapy, all participants were provided with consent forms and agreed to the following statement:

*At times*,* the data collected from questionnaires may be anonymised and used to evaluate the effectiveness of the work we offer*,* which may also contribute to published research. On these occasions*,* no personal information will be used*,* and only numerical data provided from the questionnaires will be included. This data will be collected with other anonymised data collected within the service.*

### Service and Service Users

The service evaluation took place at Chrysalis Associates within the UK, Chrysalis Associates operates across four counties within the North of England and specializes in developmental trauma and attachment for looked after and adoptive children. Commissioning for this service usually takes place through the local authority making applications to the Adoption and special guardianship support fund with each referral being made by each family’s social worker.

Data for this pilot study was obtained from service users that met each aspect of the eligibility criteria. The eligibility criteria is stated as being the following: (1) families who were showing signs of attachment difficulties as measured by the Marshak Interaction Method (Hitchcock et al., 2008), (2) had received a therapeutic package between March 2019 and March 2024, (3) had children who were showing signs of experiencing developmental trauma as measured by a pre-therapy clinical interview with the adoptive parents, and with their caregivers, (4) had a child that was suspected of having ADHD, was awaiting an assessment for ADHD, or had a formal diagnosis of ADHD (*N* = 14). For part of the analysis, each child in the ADHD group was matched to a child without diagnosed or suspected ADHD from the same dataset (who met the other inclusion criteria) based on age and gender.

Prior to entering the service all families engaged in a 2-hour developmental history and clinical intake interview appointment with a highly specialist trauma and attachment therapist, overseen by a supervising clinical psychologist. Within this appointment a holistic view of the child was ascertained from conception to present day experiences, possible comorbidities were identified and evaluated in the context of the child’s whole life story and their previous and current behavioural phenotype. From this clinical interview a decision was made as to whether the child would be eligible for inclusion in the ADHD group or a Non-ADHD group. Children met the threshold for suspected ADHD if within the clinical intake interview their parents or carers discussed a high level of ADHD symptomatology. Each child’s clinical intake interviews report was reviewed by the first author in consultation with the sixth author, and group allocation (ADHD or non-ADHD) was determined based on the overall clinical evidence available from parents and carers at intake. Children who did not meet this threshold were allocated to the non-ADHD group. All families were theraphy seeing adoptive, special guardianship, or foster families who had been able to attend and meaningfully engage in a short but intense packages of trauma and attachment therapy.

The ADHD group sample was 50% male, 85.71% White British, and was aged 5 to 12 years old with the average age at pre-therapy being 8.79 years (*N* = 14, M = 8.79, SD = 1.70). Within the ADHD sample all 14 children had experiences of developmental trauma and attachment disruption, one had spina bifida, and one had confirmed prenatal alcohol exposure. (Table 1).

**Table 1:** Sample demographic information

Age (Years)	ADHD Group (*N* = 14)	Non-ADHD Group (*N* = 14)	Between-group differences
*Mean (SD)*	8.70 (1.70)	8.70 (1.20)	*t*=0.19, *p*=.86
*Range*	5–12	5–12	
Assigned Sex			*χ²*=0, *p* = 1
*Male*	50%	50%	
*Female*	50%	50%	
Ethnicity			*χ²*=0.85, *p*=.65
*White British*	12	10	
Legal Status			*χ²*=3.72, *p*=.29
*Adopted*	10	9	
*Special Guardianship Order*	4	2	
*Foster care*	0	2	
*Placement order*	0	1	
Attendance Rates			*t*=0.43, *p*=.67
*Mean (SD)*	97.36% (7.23)	96.14% (7.38)	
*Range*	73%−100%	80%−100%	

### Neurocollaborative Therapeutic Practice

The treatment approach implemented by Chrysalis Associates builds upon Perry’s (2009) neurosequential model of therapeutics by introducing and abiding by the principles of neurocollaborative therapeutic practice. Neurocollaborative therapeutic practice builds upon the neurosequential model as instead of utilising psychotherapeutic interventions to target the brain stem, followed by the limbic brain areas, followed by the cortical brain areas in a sequential manner; neurocollaborative therapeutic approaches aim to address various brain regions concurrently with the aim of better meeting the therapeutic needs of the child and family in the therapy room. Subsequently, the therapeutic package involves the following component; (1) parent only therapeutic parenting sessions; (2) age-appropriate sensory regulation sessions; and (3) family therapy session centered around the principes of Theraplay^®^. Chrysalis Associates implements all three prongs at the same time; thus, this package is termed as being neurocollaborative, distinguishing it from the more commonly practiced neurosequential approach. Neurocollaborative approaches to therapeutic practice have been previously evaluated and found positive results (Hunsley et al., 2021; McCullough & Mathura, 2019; Purrington et al., 2022).

The therapeutic model most commonly consists of a total of 30 h of therapeutic work which are divided equally across the three prongs; 10 h are dedicated to a therapeutic parenting parent-only session; 10 h are dedicated to an age-appropriate sensory regulation program which was either delivered to the child only, parent and child, or parents only according to the presenting needs of the family unit; and finally, 10 h are dedicated to a family therapy session, which is informed by the principles of Theraplay ^®^ and takes place between the parent/s and child.

The original format for this model was to deliver the 30 sessions over a 10-week timeframe, with a weekly commitment of 3 h a week and the sessions were to be completed at a 1:1:1 ratio. However, during the timeframe the data was collected the Covid-19 pandemic occurred, which allowed families to gain more autonomy in how they received their therapeutic package as a direct result of the rise in demand for an online delivery format. Therefore, during this period there was an increased flexibility in how the sessions were delivered and accessed. Various methods were used to deliver the therapy sessions including virtual meeting platforms, in person, or a combination of the aforementioned methods. However, most commonly, the parent sessions were delivered through online videoconferencing platforms, and the sensory regulation and family sessions were delivered in person.

Each family’s therapeutic package began with the therapist tailoring the neurocollaborative therapy program to align with the family’s personal goals. However, broadly speaking, the intervention seeks to improve the attachment bonds between the parent and child, develop self-regulation skills, and decrease the levels of psychological suffering found within the family unit.

#### Therapeutic Parenting Sessions

A total 10 h of the total 30 therapeutic hours were dedicated to 10 therapeutic parenting sessions informed through the concepts of Theraplay parent sessions and Dyadic Developmental Parenting, delivered to the parents only. The sessions explored an extensive scope of psychoeducational components centered around the Theraplay approach, PACE approach (Playfulness, Acceptance, Curiosity, and Empathy), and the concepts of therapeutic parenting. Each session included a review which assessed the previous family therapy sessions, allowing capacity to enhance reflective practice. The reflective practice allowed for an in-depth exploration of the parents’ understanding of their own attachment histories and how they may impact upon the child’s behavioural presentation, while also allowing for the development of parental emotional well-being strategies.

#### Theraplay^®^-Informed Sessions

Another 10 h of the 30 prescribed hours was dedicated to 10 sessions of family therapy, based on the principles of Theraplay. These sessions involved the caregiver (s) and their child working alongside their trauma and attachment therapist. The Theraplay-informed sessions utilised activities tailored to the family’s needs within the dimensions of engagement, nurture, structure and challenge (Booth & Jernberg, 2010), whilst simultaneously prioritising the nine core conditions of Theraplay-informed practice: playfulness, coregulation, cultural sensitivity, guided by the adult, responsive attuned empathetic and reflective, interactive and relationship based, direct here-and-now experiences, multisensory, and working at the preverbal social right brain level of development.

#### Sensory Regulation Sessions

The final 10 h were dedicated to the sensory regulation therapy sessions. These sessions were developed by an Occupational Therapist and focused on supporting families in developing a greater awareness surrounding the areas they feel to be influencing their child’s ability to experience co- and self-regulation. The sensory regulation program was developed utilizing principles from The Just Right State Program (Hughes & Bhreathnach, 2018 online pdf pages 18–24) and the Alert Program (Williams & Shellenberger, 1996; Gill et al., 2018).

The sensory regulation sessions were either delivered to the child in a 1-to-1 setting, the child and parent together, or the parent only. Sessions which took place with the child or the child and parent together consisted of various activities directed at developing co-regulation and self-regulation such as utilising the language of the ‘speedometer’ to determine whether children were high engine revs, low engine revs, or just right engine revs, ‘snack and chat’ using regulating food, and a body-based attachment and regulation programme. At times a story-book series called The Scared Gang, developed by Bhreathnach (2008), was utilised. This book series aimed to introduce children to differing survival responses and sensory approaches to self-regulation. Additionally, age-appropriate mindfulness activities were also completed during these sessions. When the sensory regulation sessions were completed to parents only, the parents would attend a psychoeducational group based on the parent sessions from the Alert Program.

### Procedure & Data Collection

To evaluate the effectiveness of the therapeutic package, a pretest and post-test design was implemented for both the group of children in the ADHD cohort and the group of children in the non-ADHD control group cohort. Before starting therapy, all families completed a pre-assessment no more than 3 months before therapy began. After the pre-assessment, families commenced their therapeutic package with their trauma and attachment therapist. Trauma and attachment therapists were trained to a minimum standard of Level 1 in Theraplay with the majority of therapists being trained to a Level 2 standard in Theraplay and Dyadic Developmental Psychotherapy and also being qualified Eye Movement Desensitisation and Reprocessing therapists. All therapists were supervised by either a consultant clinical psychologist or an experienced clinical psychologist. Fidelity checks for the competence and coherence of Theraplay delivery were not routinely conducted, however case discussions took place within supervision which took place weekly, fortnightly, or monthly depending on the experience level of the therapist. During these case discussions intervention fidelity was explored when necessary.

Once completed, the post-therapy assessment measures were administered during the second to last Theraplay-Informed and therapeutic parenting sessions, with the families being informed to return the completed measures within a 2-week timeframe. The assessment measures were the Assessment Checklist for Children (ACC), the Behaviour Regulation Inventory for Executive Functioning 2nd edition parent form (BRIEF-2), the Child Behaviour Checklist (CBCL), and the Trauma Symptom Checklist for Young Children (TSCYC).

### Outcome Measures

Four outcome measures were utilized to evaluate therapeutic outcome. The Assessment Checklist for Children (ACC; Tarren-Sweeney, 2007), the Behaviour Rating Inventory of Executive Function – 2nd Edition (BRIEF-2; Gioia et al., 2015), the Child Behaviour Checklist (CBCL; Achenbach & Rescorla, 2001), and the Trauma Symptom Checklist for Children (TSCYC; Briere, 2005). An overview of these measures is presented in Table 2.

**Table 2:** Summary of outcome measures used

Measure	Description	Psychometric Properties
Assessment Checklist for Children (ACC)	A parent/carer report tool for children aged 4–11, developed by Tarren-Sweeney (2007). It includes 120 items across ten clinical and two self-esteem scales (e.g., Total Clinical Score, Non-Reciprocal interpersonal behaviour, and Indiscriminate interpersonal behaviour). These scales assess emotional and behavioural difficulties for children in care.	High internal consistency (α = 0.70–0.96); strong factorial and concurrent validity. This was derived from a review by clinicians and foster parents, with the factorial and concurrent validity strongly supporting the ACC’s construct validity.
Behaviour Rating Inventory of Executive Function – 2nd Edition (BRIEF-2)	Developed by Gioia et al. (2015), this 63-item parent-report tool for children aged 5–18. Assesses executive functioning across three indexes (Behavioral, Emotional and Cognitive) divided among nine domains (e.g., Global Executive Composite) using a 3-point Likert scale.	High internal consistency (α = 0.79–0.97) across index scores for clinical and normative populations (Hendrikson and McCrimmon, 2019). Includes three internal validity scales; Infrequent Responses, response Consistency and Negative Nature (Hendrikson and McCrimmon, 2019).
Child Behaviour Checklist (CBCL)	Developed by Achenbach & Rescorla (2001), this parent-report tool assesses emotional and behavioural problems in children and adolescents. Includes eight syndrome scales (e.g. Rule-Breaking Behaviour, Oppositional Defiant Problems and Total problems). Scales form two broad-band scales (Internalising, Externalising) and uses a 3-point Likert scale.	The CBCL exhibits high reliability with its correlation being reported at 0.93 to 1.00 (Achenbach & Rescoria, 2001).
Trauma Symptom Checklist for Children (TSCYC)	Developed by Briere (2005), this 90-item parent-report tool for children aged 3–12 assesses trauma-related symptoms across nine clinical subscales (e.g., Anxiety, Depression, PTS-Arousal and PTS- Total).	Briere et al. (2001) reported a strong reliability score (α = 0.87) and further exhibits concurrent validity with the CBCL (Wherry et al., 2008). The TSCYC includes two internal validity scales measuring under-reporting and over-reporting of symptoms (Briere, 2005).

### Data Analysis

The aim of this pilot study was to assess whether the intervention was associated with pre-post improvements for the children with ADHD, and whether any improvements were comparable for children with ADHD and children without ADHD. First, we assessed whether mean scores on each outcome crossed clinical boundaries from pre to post measures in the ADHD group (descriptive analysis). Next, we conducted paired samples t-tests to assess for any significant changes from pre to post test in the ADHD group. Finally, we conducted analyses of covariance to compare changes from pre to post-test between the ADHD and non-ADHD groups using pairs matched by age and gender. Post scores were entered as the dependent variables, group as the independent variables, and pre scores as covariates. Given the small samples, effect size was seen as being more important than statistical significance although both are reported here.

## Results

### Sample Sizes

This evaluation included 14 matched pairs of participants, accounting for a total of 28 individuals matched on the characteristics of age and gender. The sample sizes for each outcome measure varied across pre- and post-therapy assessments due to age of children; caregivers were not asked for data on children who fell outside of the specified age range for each measure. A total of 13 families completed the TSCYC at pre-therapy, and 12 families completed the post-therapy assessment. A total of 11 families completed the CBCL at pre-therapy and 10 families completed the post-therapy assessment. The BRIEF-2 assessment was completed by 12 families at both time points and the ACC was completed by eight families at both time points.

### Outcomes Measured

Descriptive analysis was applied to all scales across all four outcome measures. Statistical analysis was applied to only: the TSCYC subscales of Anxiety, Depression, Post-Traumatic Stress (Arousal), and Post-Traumatic Stress Total; the CBCL subscales of Total Problems, Rule-Breaking Behaviour, and Oppositional Defiant Problems; the BRIEF2 scale of Global Executive Composite; and the ACC subscales of Total Clinical Score, Non-Reciprocal interpersonal behaviour, and Indiscriminate interpersonal behaviour.

### Descriptive Analysis

Descriptive analysis indicated that the ADHD sample presented with elevated levels of emotional, behavioural, and relational challenges at baseline relative to normative expectations. Mean pre-therapy scores across multiple TSCYC, CBCL, and BRIEF2 domains fell within the borderline or clinically significant ranges, indicating that the ADHD sample represented an acute and clinically complex cohort rather than a subthreshold or community-level presentation. Although not all domains reached clinical thresholds, the pattern of scores across measures reflected widespread challenges, providing important context for interpreting both the magnitude and clinical relevance of observed changes.

Clinically meaningful changes within the ADHD group were observed on the TSCYC scale of Depression with mean average scores reducing from a t-score of 65.2 at pre-therapy (borderline clinically significant) to a t-score of 60.6 (average) at post-therapy, and the CBCL scales of Externalising Problems which reduced from a t-score of 70 (clinically significant) at pre-therapy to a t-score of 67.9 (borderline clinically significant) at post-therapy and Withdrawn/Depressed presentations which reduced from a t-score of 66.6 (borderline clinically significant) at pre-therapy to a t-score of 61.8 (average) at post-therapy. Mean average score reductions were also observed across seven of the nine TSCYC scales, 12 of the 17 CBCL scales, four of the 13 BRIEF-2 scales, and six of the 15 ACC scales, see supplementary material one for raw score data from the ADHD group.

### Tests of Normality

Shapiro-Wilk tests were used to assess the normality of the data distributions for all outcome variables. Results indicated that all data were normally or approximately normally distributed, therefore supporting the use of parametric statistical tests.

### Paired t-tests (One-sided)

Paired t-tests (one-sided) were conducted to examine changes in all outcomes from pre- to post-test in the ADHD group. The results showed a significant reduction on the CBCL scales of total problems, rule breaking behaviour, and oppositional defiant problems and on the TSCYC scale of anxiety. These statistically significant changes each reported a medium effect sizes (see Table 3). No other significant changes were found.

**Table 3:** Descriptive statistics and results of paired t-tests (1-sided) for pre and post test scores in ADHD group

Outcome measure	Pre M (SD)	Post M (SD)	Difference	t(df)	*p*	d
ACC-TCS	59.75 (7.29)	58.88 (8.87)	− 0.87	1.27 (21)	0.11	0.27
ACC-NR	55.63 (8.78)	56.88 (8.01)	+ 1.25	1.33 (21)	0.10	0.28
ACC-Ind	55.88 (8.11)	51.63 (7.41)	− 4.25	1.17 (21)	0.13	0.25
BRIEF-GEC1	72.58 (10.17)	72.67 (8.76)	+ 0.09	0.14 (23)	0.44	0.03
CBCL-TP	72.40 (4.84)	70.10 (7.51)	− 2.30	2.52 (23)	0.01**	0.51
CBCL-RBB	64.40 (7.34)	63.30 (6.52)	− 1.10	1.79 (23)	0.04*	0.37
CBCL-ODP	68.50 (9.03)	66.6 (9.14)	− 1.90	1.92 (23)	0.03*	0.39
TSCYC-TotPTS	66.77 (17.55)	68.85 (20.61)	+ 2.08	0.16 (24)	0.44	0.03
TSCYC-Anx	62.00 (18.21)	57.54 (15.80)	− 4.46	2.67 (24)	0.007**	0.53
TSCYC-Dep	65.23 (17.68)	60.69 (18.59)	− 4.54	1.68 (24)	0.053	0.34
TSCYC-ArPTS	71.08 (18.46)	70.31 (17.47)	− 0.77	1.33 (24)	0.10	0.27

ACC = Assessment Checklist for Children; BRIEF = Behaviour Rating Inventory for Executive Function; CBCL = Child Behaviour Checklist; TSCYC = Trauma Symptom Checklist for Young Children; TSC = Total Clinical Score; NR = Non-Reciprocal; Ind = Indiscriminate; GEC1 = Global Executive Composite; TP = Problems; RBB = Rule Breaking Behaviour; ODP = Oppositional Defiant Problems; TotPTS = Total Post-Traumatic Stress; Anx = Anxiety; Dep = Depression; ArPTS = Arousal Post-Traumatic Stress

* *p*<.05, **p<.01

### Analysis of Covariance

Analyses of Covariance (ANCOVA) were conducted to evaluate differences in pre- to post-therapy improvements between participants with ADHD and their matched controls across all outcome measures. Each ADHD participant was matched to a control participant based on age and gender. The dependent variables for the analysis were the post-test scores for each outcome measure, and the corresponding pre-test scores as covariates, with the group (ADHD vs. Non-ADHD control) as the fixed factor. The ANCOVA results revealed no statistically significant group differences on any of the outcome measures after baseline scores had been controlled for (all *p*’s > 0.05).

### Summary of Findings

The analyses revealed significant reductions in reported behavioural and trauma-related anxiety symptoms from pre- to post-therapy, as measured by the CBCL and the TSCYC for the ADHD group. Paired *t*-tests indicated medium effect sizes for these reductions. Additionally, no significant between-group differences were identified in the ANCOVA analysis after controlling for baseline scores and matching for age and gender.

## Discussion

This pilot study aimed to review the efficacy of a 30-hour neuro-collaborative Theraplay-informed psychotherapeutic intervention on the presentations of developmental trauma and attachment difficulties in looked after children with ADHD compared to a match-controlled group of children with presentations of developmental trauma and attachment difficulties but without ADHD.

Analysis of trauma, emotional, and behavioural health revealed significant improvements in the ADHD group from pre-test to post-test scores on the CBCL scales of total problems, rule breaking behaviour, and oppositional defiance and on the TSCYC scale of anxiety. Additionally, clinically meaningful changes were observed on the CBCL scales of externalising problems and withdrawn/depressed presentations and the TSCYC scale of depression. Moreover, there were no significant differences demonstrated between the pre and post therapy measurements of the ADHD group and their matched non-ADHD controls on any of the outcome variables. The overall findings suggest that the 30 h neuro-collaborative Theraplay-informed intervention shows promise for families with children with ADHD and more significantly, the intervention was of comparable effectiveness for families with looked after children who have experienced developmental trauma and attachment disruptions and have a diagnosis of ADHD as it was for families with looked after children who have experienced developmental trauma and attachment disruptions, without a diagnosis of ADHD.

### Trauma Symptoms

The TSCYC scores showed statistically significant improvements in the Anxiety domain and clinically meaningful improvements in the Depression domain, following therapy.

From a cognitive perspective of PTSD, symptoms such as anxiety and depression commonly occur as a result of the re-occurrence of past problematic memory, the meaning that is applied to these memories and the traumatic experiences, and the maintenance behaviours undertaken to cope with such experiences (Ehlers & Clark, 2000). Additionally, affect regulation is a common presentation in individual who have experienced complex PTSD (Stubley et al., 2025). Moreover, ADHD is increasingly being understood as a divergent condition of regulatory systems as opposed to purely attentional systems (Barkley, 1997; Christiansen et al., 2019), therefore for children who have experiences of developmental trauma, attachment disruption, and a diagnosis of ADHD their presentations of trauma related affect may be even more pronounced (Thorell et al., 2012). Therefore, this poses as a difficulty for clinicians to devise treatment plans best suited for children who have ADHD and experiences of developmental trauma and attachment disruptions due to their intersectionality that may exacerbate presenting symptomology.

Despite these challenging aspects of intersectionality, the results of this pilot study are promising as the Theraplay-informed intervention was shown to reduce trauma-related affect dysregulation in the domains of anxiety and depression for children within the ADHD group. Although the present study did not independently measure the differential impact of individual intervention components (e.g., Theraplay-informed activities versus parenting support versus sensory regulation sessions), changes were observed specifically in trauma-related anxiety symptoms and depressive affect. Each of these domains are closely linked to attachment security and co-regulatory processes and therefore these findings are consistent with existing evidence that Theraplay-informed interventions support emotional regulation through relational mechanisms.

A core focus of Theraplay-informed practice is to utilise activities to foster closeness and greater security within the parent-child attachment relationship, with previous research demonstrating that Theraplay can successfully increase closeness within interpersonal relationships (Weir et al., ; Wettig et al., 2011). Additionally, Theraplay-informed practise prioritises principles of co-regulation and is multi-sensory to facilitate sensory regulating experiences to support children. Again, previous research has demonstrated the impact of Theraplay in the development of behavioural and emotional regulation (Francis et al., 2017; Lindaman & Lender, 2009; Rezaeianzadeh & Yazdanfar, 2024; Siu, 2009). Taken together, these findings (the observed improvements in trauma-related affect dysregulation in the domains of anxiety and depression) are theoretically aligned with the relational and co-regulatory mechanisms emphasised within Theraplay-informed practice and may help explain the observed reductions in trauma-related affective symptoms. This notion is supported by Thorell et al. (2012) which concluded that attachment disruptions significantly exacerbated ADHD symptoms in children, independent of conduct problems and executive functioning. Therefore, interventions that look to strengthen attachment bonds are likely to be impactful for this cohort. However, future studies employing component analyses or session-level fidelity measures would be required to determine the relative contribution of Theraplay-informed activities, sensory regulation input, and parenting support to observed affective change.

Additionally, the observed reductions in trauma-related anxiety and depressive affect may also be understood within the context of the intervention’s parenting support components, although the individual contributions of each therapeutic aspect were not independently measured in the present study. Previous research has found that integrating behaviour management training to parents alongside Theraplay-informed practice was effective in reducing the symptoms of ADHD (Askari et al., 2014). The therapeutic parenting sessions within this intervention aimed to support caregivers to pick apart, understand, and isolate their child’s unique ADHD behavioural presentations from their unique trauma behavioural presentations and therefore theoretically align with the mechanisms proposed by Askari et al. (2014). Taken together, the findings of Askari et al. (2014) and the present study are consistent with the potential value of a multi-modal approach in which attachment and trauma informed relational work is delivered alongside parent-mediated behavioural and environmental support for families with children who have experienced developmental trauma, attachment disruption, and have a diagnosis of ADHD. This integrated framework aligns with neurodevelopmental conceptualisations of ADHD as a whole brain-based divergence (Rubia, 2018), and highlights the need for bespoke, varied and integrated interventions.

Conversely, there were no significant changes noted in the domains of Post Traumatic Stress Arousal nor the Post-Traumatic Stress Total which this domain contributes to. The symptomology associated with the arousal domain includes concentration difficulties, hypervigilance, and irritability due to an exaggerated sympathetic nervous system and the ‘fight, flight or freeze’ response (Shiromani et al., 2014). Whilst these symptom clusters are strongly associated with trauma; presenting with concentration, attentional focus, and distractibility (which can look like hypervigilance) difficulties are core components of ADHD and have neurobiological underpinnings (Purper-Ouakil et al., 2011; Tucha et al., 2006). Therefore, it was not anticipated that the items associated with post-traumatic stress arousal domain on the TSCYC assessment tool, which are heavily orientated around the nervous system and attentional focus, would be likely to show change in the context of children with ADHD.

### Behavioural Presentations

The CBCL revealed statistically significant reductions in total problems, rule-breaking behaviour, and oppositional defiant behaviour, alongside clinically meaningful changes in externalising and withdrawn/depressed presentations. These findings indicate that the Theraplay-informed intervention was associated with improvements in caregiver-reported behavioural and emotional functioning in children with histories of developmental trauma, attachment disruption, and ADHD. While the present study did not directly assess proposed mechanisms of change, existing attachment-based and parenting intervention literature offers a useful framework for interpreting these findings.

One possible formulation underpinning rule-breaking and oppositional defiant behavioural presentations in this cohort is that these children may have developed a negative internal working models resulting from their early life which informs them that: (1) they are not worthy of being kept safe, (2) others around them will not keep them safe, and (3) the world is generally an unsafe place (Bowlby, 1969, 1973, 1980, 1982, 1988). Therefore, rules are broken and an oppositional stance is taken to primary caregivers in order for the young people to ascertain a sense of control and safety. An alternative formulation within this context is that children with ADHD can appear to break rules and present as oppositional if the environment around them does set them up to successfully manage their ADHD symptoms of impulsivity and inattentiveness. For the children within this cohort, it is possible these formulations may co-occur and are not mutually exclusive.

Theraplay emphasises the dimensions of structure and engagement, which have been associated in prior research with improvements in behavioural regulation and caregiver–child interactions (Purrington, Lindaman, et al. 2025). The structure dimension aims to increase children’s experiences of predictability and safety through repeated, adult-led interactions, while engagement promotes experiences of being seen, valued, and emotionally connected (Norris & Lender, 2020). Previous studies have suggested that children with regulatory difficulties, including those with trauma histories and neurodevelopmental conditions, particularly benefit from increased environmental structure (DiPasquale, 2009). Although these dimensions were not independently measured in the present study, the observed reductions in rule-breaking behaviour are consistent with literature indicating that structured, relationally focused interventions can increase behavioural regulation by enhancing perceived safety and relational trust.

Reductions in oppositional defiant behaviours may also be understood in relation to attachment-based processes such as co-regulation and caregiver responsiveness. Theraplay’s nurture dimension emphasises caregiver-led soothing and physical and emotional regulation experiences (Norris & Lender, 2020), which parallel mechanisms identified in other attachment-based interventions. For example, Manafi et al. (2020) reported reductions in oppositional behaviours following attachment-focused psychotherapy, attributing change to improvements in parental responsiveness and reductions in parenting stress. Again, while attachment security and co-regulation were not directly assessed in the present study, the pattern of CBCL change observed aligns with these previously identified therapeutic processes.

Additionally, it is also possible that the therapeutic parenting aspect of the intervention supported parents to better understand their child’s ADHD, trauma, and attachment related presentations. These therapeutic parenting sessions were informed by the principles of dyadic developmental parenting from the model of dyadic developmental psychotherapy (DDP) and Theraplay parent sessions. Previous studies exploring the use of DDP for looked after children have found that the approach can support caregivers to cultivate a greater understanding of their child’s behavioural and emotional world (Agbayani, 2014; Turner, 2012; Wingfield & Gurney-Smith, 2019), additionally DDP-informed therapeutic parenting, parent-only consultations have also been indicated to enhance parental understanding (Gosling & Purrington, 2023) whilst also supporting the needs of caregivers (Shepherd et al., 2023). Moreover, caregiver-only Theraplay-informed support sessions have also been found to increase caregiver perception of their parenting skills and knowledge and to have a positive impact on caregivers (Purrington, Hartley, et al. 2023). Whilst important to acknowledging that changes in caregiver perception were not independently measured, these findings provide a plausible context for understanding the reductions in caregiver-reported behavioural difficulties observed within this study.

### Regulation & Executive Function

Analysis of the BRIEF2 scores indicated that there was no significant reduction in the Global Executive Composite (GEC) for children in the ADHD group following the intervention. Contemporary theoretical models conceptualise ADHD not as a transient behavioural difficulty, but as a neurodevelopmental condition characterised by enduring impairments in executive functioning and self-regulation (Brown, 2013). From this perspective, executive functions represent higher-order cognitive control processes that develop gradually over time and are strongly influenced by neurobiological factors.

In this context, the absence of significant change in the GEC is theoretically coherent. The BRIEF2 GEC captures broad, trait-like aspects of executive functioning, including inhibition, working memory, planning, and emotional control, which are core features of ADHD and are relatively stable across development. As such, the present findings suggest that while the intervention may have supported improvements in affect regulation and emotional wellbeing, it did not directly alter underlying executive functioning capacity as measured by the BRIEF2. This distinction is important when interpreting treatment outcomes for children with ADHD, particularly those who have also experienced developmental trauma and attachment disruption. As opposed to targeting executive functioning capacity itself, the intervention may have exerted its effects through environmental and relational pathways, including enhanced caregiver attunement, co-regulation, and behavioural scaffolding. Supporting caregivers to modify environmental demands and respond more effectively to emotional and behavioural cues may reduce the functional impact of executive difficulties (as reflected in findings on the CBCL) without necessarily changing executive functioning at a structural level (as reflected in findings on the BRIEF2 GEC). This finding therefore has important implications for clinical practice, suggesting that therapeutic work with looked after children with ADHD may be more appropriately focused on supporting regulation through relational and environmental adaptation, rather than aiming for direct remediation of executive functioning skills. Such an approach may also help to establish realistic expectations among caregivers and clinicians regarding the anticipated outcomes of attachment- and trauma-informed interventions.

Separately, it is also important to consider that this study evaluated the use of the therapeutic intervention on ADHD as one whole concept, as opposed to the effects on the intervention on the differing subtypes of ADHD. There are three subgroups under the ADHD umbrella, this study cannot provide any indication as to whether the impact of the Theraplay-informed intervention is mediated by the type of ADHD a child has. One previous study reports two investigations that reveal significant increases in the attention span of shy and socially withdrawn children following Theraplay (Wettig et al., 2011). Suggesting that perhaps Theraplay-informed practice could be effective for supporting children with inattentive subtype ADHD. However, at present much greater evidence is required to determine this connection and further to determine whether the subtype of ADHD effects therapeutic outcomes related to regulation and executive functioning in looked after children with ADHD.

### Attachment Presentations

The ACC indiscriminate scale relates to a child’s tendency to form overly close relationships without considering appropriate boundaries and therefore these children may engage in attention-seeking behaviour with unfamiliar adults, act too friendly with strangers or change friends quickly. The ACC non-reciprocal scale refers to the quality of interpersonal skills and behaviours, characterised by lack of empathy, appearing uncaring, or presenting as manipulative. There were no significant reductions found on the ACC scales of total clinical score, indiscriminate and non-reciprocal.

The lack of a statistically significant change between pre- and post-therapy scores on the indiscriminate scale invites cautious consideration. Indiscriminate behaviour has been observed in some looked after populations regardless of the presence of a preferred attachment figure, suggesting that indiscriminate behaviours may occur independently of attachment security (Zeanah et al., 2002). Within the present cohort, the persistence of indiscriminate behaviours following therapy may reflect influences beyond attachment-related processes. One possible speculation relates to difficulties in executive functioning, particularly inhibitory control. Impairments in inhibitory functioning have been shown to be characteristic of ADHD and are less strongly associated with other co-occurring conditions such as mood or anxiety disorders (Barkley, 1999). Behaviourally, reduced impulse inhibition may manifest as socially indiscriminate interactions. However, this interpretation remains speculative and highlights an area for future research.

Moreover, the absence of a statistical change on the non-reciprocal scale may have been associated with the overshadowing of inattentive subtype ADHD presentations. Inattentive presentations which include: appearing not to listen when spoken to directly, failing to follow through on instructions, having difficulty focusing, forgetting things often, and being easily distracted (APA, 2013), may be misinterpreted by caregivers as children choosing to behave this way and therefore presenting as uncaring, suspicious, secretive, and potentially non-empathic and manipulative depending on the internal narrative the caregiver is applying to the behavioural presentation. Presenting as uncaring, suspicious, secretive, non-empathic, and manipulative are all items used to assess for non-reciprocal behaviour (Tarren-Sweeney, 2007). Therefore, it is possible that a lack of significant changes on this scale provides evidence for ineffective measurement tools as opposed to the ineffectiveness of the intervention.

It is also possible that the absence of significant change on the ACC scales reflects a genuine lack of therapeutic change. The intervention evaluated is considerably brief (30 h often delivered over 10 weeks). Entrenched interpersonal behaviours such as indiscriminate and non-reciprocal patterns may therefore require longer-term interventions to demonstrate measurable and meaningful change. In conclusion, the absence of statistically significant change on the ACC scales within the ADHD group cannot be used to decisively state that this population of children and their carers did not feel more connected or attuned to one another following intervention. More likely perhaps, these findings highlight the significant overlap between ADHD presentations and developmental trauma presentations or limitations in the brevity of the intervention period, and therefore it is difficult to ascertain significant attachment-related changes within an ADHD cohort without an improvement in the available outcome measures or lengthening the intervention period.

### Benefits of a Novel Approach

It is understood that current practices in behavioural parent training typical involve parent coaching within child-directed play. Such coaching takes place as early as the first session. However, the present intervention differs in both intensity and scope. Rather than focusing primarily on behavioural management strategies through play, the Theraplay-informed neurocollaborative therapeutic model integrates concurrent parent-only therapeutic parenting sessions, sensory regulation interventions, and attachment-focused family therapy informed by Theraplay. This multi-pronged approach targets relational, regulatory, sensory, and neurodevelopmental processes simultaneously, reflecting a broader therapeutic focus compared to traditional behavioural parent training. The intervention outlined within this paper embeds parent coaching and support within an attachment and trauma informed framework that emphasises co-regulation, caregiver reflective capacity, and emotional attunement. Considering the finding presented within this study, the Theraplay-informed neurocollaborative approach which concurrently brings together parent-only therapeutic support, child-focused sensory regulation support, and attachment-focused family therapy for parent-child dyads may offer added value for children presenting with complex regulatory and relational difficulties such as children with histories of developmental trauma and attachment disruption, alongside presentations or diagnosis of ADHD.

### Limitations

This study presents with limitations that possibly undermine the reliability and validity of the findings, and it is important to acknowledge these. Most significantly, the research design poses as a limiting factor due to the high number of variables and relatively low sample size, reducing the statistical power and potentially obscuring significant effects. Furthermore, the lack of a control condition, comparing treatment and non-treatment groups, contributes to the risk of placebo and regression to the mean effects within the dataset. This limits our interpretation of the true effect sizes delivered by the intervention.

Additionally, the relatively small sample size (*n* = 14 per group) increases the possibility of spurious effects. Small samples are more susceptible to inflated effect sizes and reduced statistical power, limiting the generalisability of results. Although the matched-pairs design strengthens internal validity, findings should be interpreted cautiously and considered preliminary until further larger adequately powered samples are studied.

Not all children within the examined population had a formal clinical diagnosis of ADHD, with almost half of the sample only suspected to have ADHD. Moreover, subtype distinction was not evident, even in individuals meeting clinical diagnostic criteria for ADHD. This limitation makes it difficult to determine whether the observed statistically significant outcomes can be reliably generalised to the wider population of looked after children, who do present with a formal clinical diagnosis of ADHD.

Moreover, despite trauma and attachment therapists working in line with the core therapeutic modalities and under supervision of a clinical psychologist, fidelity checks were not routinely conducted, and therefore the quality of therapeutic practice cannot be confirmed. This, again, may obscure the interpretation of findings around the efficacy of the Theraplay-informed intervention as delivered for the current cohort.

Finally, data collection for this study spanned a five-year period, during which the Covid-19 pandemic occurred. Pandemic-related stressors, disruptions to routines, and changes in service delivery may have influenced children’s baseline presentations, engagement in therapy, and/or responsiveness to the intervention. Although core intervention components remained consistent, variability in contextual factors such as a fully in person delivery format versus a mixed delivery format of remote telehealth sessions and in person sessions, increases in acute caregiver stress, and environmental instability, may have each contributed to heterogeneity in outcomes. Additionally, over and above the Covid-19 pandemic, further unmeasured and unaccounted for factors such as the rising cost of living between 2019 and 2024, changing demands on services, and/or clinician experience may have influenced results. These temporal and contextual variables were not controlled for and represent a further consideration when interpreting findings.

### Implications for Research and Practice

The present study highlights that the neuro-collaborative Theraplay-informed intervention was just as effective in obtaining positive outcomes on trauma, behavioural, and emotional measures in looked after children with developmental trauma, attachment disruptions, and ADHD symptomology in comparison to their non-ADHD counterparts. It is hoped that this study can provide tentative, provisional support for the clinical application of Theraplay-informed practice, therapeutic parenting, and sensory therapy for children similar to the cohort examined within this study. As the literature base develops with larger scale cohorts and more varied research designs, services and practitioners working with looked after children with ADHD symptomology could consider ongoing training for the application of similar Theraplay-informed practices, thus ensuring continued high standards of the available therapeutic provisions for families that seek this.

To gain further insights, qualitative research involving therapists who work with families of looked after ADHD children would be useful to explore their experiences of adapting Theraplay-informed practice, sensory regulation work and therapeutic parenting to support their model’s efficacy with the ADHD population. This is particularly relevant for the looked after population, whose intersectionality is often complex and confounding within the existing literature base. It is equally imperative to hear the lived experiences of this intervention directly from the families, including carers, as well as the children themselves.

Finally, adapting the limitations of the present study for future quantitative research into Theraplay-informed practice with a larger looked after ADHD cohort compared to a non-treatment control may help to provide a clearer picture on its efficacy. This is also vital in disaggregating the effects of a formal clinical diagnosis of ADHD within the population set against the currently confounding aspects of the intersectionality explored within this piece of work.

## Conclusion

This pilot study aimed to review the efficacy of 30 h neuro-collaborative Theraplay-informed psychotherapeutic intervention on presenting difficulties of trauma and attachment in looked after children with ADHD who have experienced developmental trauma and attachment disruptions in comparison to their non-ADHD equivalents. The findings revealed statistically significant improvements on the scales of total problems, rule breaking behaviour, and oppositional defiant problems on the CBCL, and anxiety on the TSCYC. Additionally, clinically meaningful improvements were reported on the scales of externalising problems and withdrawn/depressed presentations on the CBCL and on the depression scale on the TSCYC. However, there were no statistically significant or clinically meaningful improvements noted when comparing pre and post measures of executive functioning and attachment between the ADHD group and their matched controls.

## Supplementary Information

Below is the link to the electronic supplementary material.

Supplementary File 1 (XLSX 18.7 KB)

## Data Availability

We have included a supplementary file showing the anonymised raw data for the ADHD group.
